# Focal Adhesion Kinases in Adhesion Structures and Disease

**DOI:** 10.1155/2012/296450

**Published:** 2012-07-19

**Authors:** Pierre P. Eleniste, Angela Bruzzaniti

**Affiliations:** Department of Oral Biology, Indiana University School of Dentistry, DS241, 1121 W. Michigan Street, Indianapolis, IN 46202, USA

## Abstract

Cell adhesion to the extracellular matrix (ECM) is essential for cell migration, proliferation, and embryonic development. Cells can contact the ECM through a wide range of matrix contact structures such as focal adhesions, podosomes, and invadopodia. Although they are different in structural design and basic function, they share common remodeling proteins such as integrins, talin, paxillin, and the tyrosine kinases FAK, Pyk2, and Src. In this paper, we compare and contrast the basic organization and role of focal adhesions, podosomes, and invadopodia in different cells. In addition, we discuss the role of the tyrosine kinases, FAK, Pyk2, and Src, which are critical for the function of the different adhesion structures. Finally, we discuss the essential role of these tyrosine kinases from the perspective of human diseases.

## 1. Introduction

The extracellular matrix (ECM) is an insoluble supra-structure comprised of a variety of matrix components including fibronectin, glycosaminoglycans, chrondronectin, osteonectin, collagens, laminin, proteoglycans, and growth factors [1–6]. The ECM provides the scaffold for cell attachment which is necessary for several diverse cellular activities, including cytoskeletal remodeling, polarization, differentiation, migration, and invasion [7–9]. Binding to the ECM is regulated by various signaling pathways that control the assembly and disassembly of three distinct, but functionally related actin and integrin-containing adhesion structures known as focal adhesions, podosomes, and invadopodia. In this review, we will discuss our current understanding of the similarities and differences between focal adhesions, podosomes, and invadopodia. We also will highlight several important tyrosine kinases and other signaling proteins that are known to control the formation and function of these adhesion structures, and we will discuss their role in pathophysiology.

## 2. Focal Adhesions 

Focal adhesion formation and turnover has been used as a model system for understanding the mechanisms of cellular adhesion. Although focal adhesions, podosomes, and invadopodia share common signaling proteins, they are distinct in cellular architecture and function (summarized in Table 1). Focal adhesions, also known as “focal contacts,” were identified over 30 years ago by electron microscopy and described as electron-dense plaques associated with actin filament bundles [10]. Focal adhesions can be considered to be large protein assembly complexes that spread mechanical forces from sites of cell adhesion to the cell body. In addition, focal adhesions regulate intracellular signaling pathways necessary for cell migration, growth, proliferation, embryogenesis, wound healing, and tissue repair [11–14]. Focal adhesions are comprised of a wide range of signaling proteins [15], such as the tyrosine kinases Pyk2 [16, 17], FAK [18, 19], Src [20, 21], Abl [22], and integrin-linked kinase [23]; the phosphatases PTP-PEST [24] and PTP1B [25]; the actin-binding proteins paxillin [26, 27], talin [23, 28–30], vinculin [23, 28–30] and tensin [31], the GTPases dynamin [32] and Cdc42/Rho [33, 34], as well as scaffolding proteins p130Cas [35] and Crk [27]. Many of these proteins have been shown to play predominantly a structural role or are involved in signal transduction [36].

 Several protein kinases are recruited to focal adhesions upon cell attachment. These protein platforms recruit adaptor proteins and lead to the activation of complex network of signaling cascades that regulate basic cellular functions [16, 36]. An important tyrosine kinase found in focal adhesions is the focal adhesion kinase (FAK). FAK is a 125 kDa cytoplasmic tyrosine kinase that is activated upon integrin engagement and controls signaling pathways crucial for cell proliferation, migration, and survival [37]. The C-terminal domain of FAK is known as the focal adhesion targeting domain (FAT). As its name implies, the FAT domain is involved in directing FAK to focal adhesion complexes in a variety of cells [38]. In contrast, the N-terminal domain of FAK is known as the FERM domain (F for the 4.1 protein, Ezrin, Radixin, and Moesin). The central kinase domain of FAK, which itself is activated by phosphorylation, directs the phosphorylation of several signaling protein such as paxillin, Grb2 and p130Cas [39]. *In vitro* studies have shown that the FERM binds directly to the intracellular domain of the *β*1-integrin subunit and regulates FAK kinase activity [40]. It was also discovered that blocking *β*1-integrin function leads to FAK dephosphorylation, which in turn increases the sensitivity of malignant tumors to ionizing radiation and delays the growth of human head and neck squamous cell carcinoma cell lines [41].

**Figure 1:**
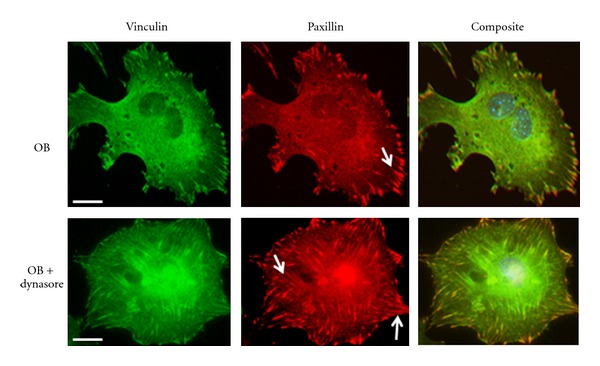
Inhibition of dynamin increases focal adhesions. Calvarial-derived osteoblasts (OBs) were treated with dynasore (90 *μ*M) or vehicle for 1 hr and labeled for vinculin (green) or paxillin (red). Green and red channels were merged to form the composite image. Scale bar indicates 10 *μ*m. Arrows show location of focal adhesions.

FAK and the tyrosine kinase Src play a central regulatory role in focal adhesion turnover, and deletion of either of these kinases increases focal adhesion stability [42]. In addition, it has been shown that FAK and Src work in concert with the GTPase dynamin to regulate microtubule-induced focal adhesion disassembly [43]. In studies by Ezratty and colleagues, FAK−/− fibroblasts exhibited reduced dynamin accumulation around focal adhesions compared to controls [43], suggesting that FAK regulates dynamin localization and recruitment to focal adhesions. In addition, Wang and others demonstrated that Src phosphorylates dynamin at tyrosine residues, which promotes the translocation of dynamin to focal adhesions by FAK [32]. Disruption of the Src-FAK-dynamin complex blocked focal adhesion disassembly and fibroblast migration [32]. Using bone-forming osteoblasts as our model system, we also found that dynamin is expressed in osteoblasts and inhibition of its GTPase activity with the chemical inhibitor dynasore, increased the number of vinculin and paxillin-positive focal adhesions in osteoblasts, compared to control cells (Figure 1). Interestingly, we found that dynamin is also localized to actin-rich podosomes, in bone-resorbing osteoclasts [44, 45]. Moreover, dynamin knockdown with shRNA or overexpression of a GTPase-inactive dynamin mutant increased podosome stability and the thickness of the podosome belt and decreased osteoclast bone resorbing activity [44]. Together, these studies reveal that dynamin's GTPase activity is necessary for both focal adhesion turnover in osteoblasts as well as podosome turnover in osteoclasts. Furthermore, these findings suggest potential similarities in the mechanism of turnover of focal adhesions and podosomes, which is likely to be dependent on the complement of signaling and scaffolding proteins present in different cell types.

## 3. Podosomes

**Figure 2:**
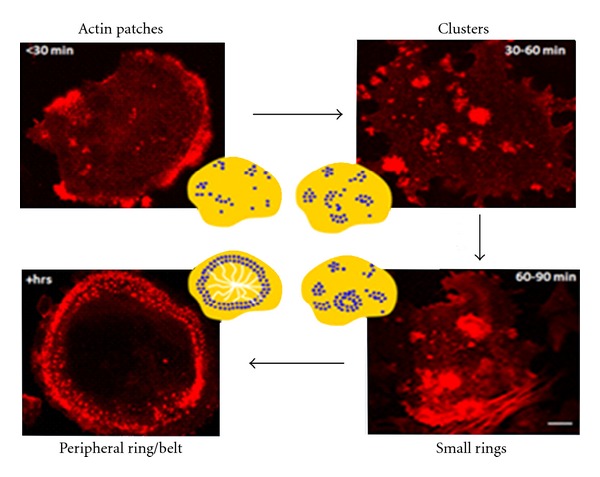
Dynamics of podosome organization in osteoclasts. Osteoclasts were generated from mouse bone marrow and plated on FBS-treated coverslips for various times. Cells were fixed and stained with rhodamine phalloidin. Actin patches are found soon after osteoclast attachment. Actin patches then reorganize into small rings and then into a peripheral podosome belt. The podosome belt is stabilized by the microtubule network. Scale bar is 10 *μ*m.

Podosomes are highly dynamic adhesion structures found in a wide variety of migratory cells including macrophages, osteoclasts, endothelial cells [46–50], transformed fibroblasts [51], and carcinoma cell lines [52]. They were first identified in the 1980s in v-Src-transformed fibroblasts [53, 54]. Podosomes and focal adhesions are both cell-matrix adhesion sites, but they differ in their structural design and turnover rates [55–59] (Table 1) despite sharing a large number of common signaling proteins, such as FAK, dynamin, talin, paxillin, Wasp, and vinculin [60]. Podosomes turnover occurs very rapidly with an apparent half-life of 2–12 min and involves the polymerization and depolymerization of the central F-actin core [50, 61]. Podosomes first appear as small actin dots which are then reorganized into small rings or rosettes with a diameter of 0.5–1 *μ*m and a depth of 0.2–0.4 *μ*m [62, 63] (Figure 2). The assembly of podosomes in macrophages and osteoclasts is dependent on an intact microtubule system [49, 64]. The central core of F-actin is surrounded by a ring of molecules that are involved in adhesion, matrix degradation, or migration. These proteins include the tyrosine kinases Pyk2 and Src [13], actin-associated proteins [51, 65], integrins [66], and their associated proteins [50], intermediate filaments [47], motor proteins [67] and metalloproteases [50, 68]. *In vitro* studies demonstrated that RhoA, Rac1, and Cdc42 are also involved in the regulation of podosomes turnover [69, 70] and perhaps in recruiting podosomes to the leading edge of cells following microtubule-dependent cell polarization [64, 69, 71–73]. 

 In contrast to focal adhesions, podosomes are found at sites of ECM degradation [51, 74]. The metalloproteases MT1-MMP and MMP-9 have been localized to podosomes, strongly supporting a role for podosomes in ECM degradation [56, 68, 75] in addition to adhesion [76]. This is well illustrated in osteoclasts, the primary bone-resorbing cells found in the body. In mature osteoclasts, podosomes are organized into a ring or belt-like structure at the cell periphery (referred to as the sealing zone) [50, 77]. This unique actin- and integrin-rich structure functions to dock osteoclasts to ECM proteins in bone and seals off the bone-resorbing compartment. This allows for the localized secretion of acidifying protons, chloride ions, and bone matrix-degrading metalloproteases [70].

 In response to integrin engagement, and in the presence of intracellular calcium, Pyk2 is autophosphorylated at tyrosine residue Y402, which is essential for its catalytic activity [37, 78, 79] and for downstream signaling via p130Cas, Src, Cbl, integrins, gelsolin, and paxillin and the tyrosine phosphatase PTP-PEST [24, 80–82]. Pyk2 is expressed at high levels in the nervous system and in various hematopoietic cells [57, 83]. Pyk2 is expressed in osteoblasts [84, 85] and osteoclasts [45, 64]. Deletion of Pyk2 in osteoblasts affects differentiation, migration, and actin remodeling [84, 85]. In osteoclasts, Pyk2 is localized to the podosome belt and deletion of Pyk2 leads to a decrease in osteoclast bone resorption, which contributes to the osteopetrotic phenotype observed in Pyk2-deficient mice [64, 84]. Whereas deletion of Pyk2 in osteoblasts affects focal adhesion turnover (our unpublished findings), osteoclasts lacking Pyk2 exhibit structurally disorganized podosomes [64]. Src has also been shown to be indispensable for osteoclast function and is necessary for podosome assembly/disassembly [86, 87]. Osteoclasts lacking Src exhibit abnormal podosome rings, resulting in a dysfunctional sealing zone [88]. Leupaxin is a member of the focal adhesion-associated adaptor proteins and has been found to be associated with the podosome-belt (sealing zone) in osteoclasts [89, 90]. It was also demonstrated that leupaxin forms a signaling complex with Pyk2, c-Src, and PTP-PEST which regulates the migration of prostate cancer cells [91]. Finally, as discussed above, the GTPase dynamin regulates podosome assembly and dynamics in osteoclasts [44, 63, 92] in a process that involves Src [44]. These studies and others demonstrate that distinct signaling proteins work in concert to regulate podosome organization and turnover in osteoclasts, and perhaps in podosome-containing migratory cells. 

 The tyrosine kinase Pyk2 is a homolog of FAK and shares 45% overall sequence identity and 60% amino acid identity within the catalytic domain. Structurally, Pyk2 also contains an N-terminal FERM domain, a central catalytic core, several proline rich domains (PRDs), and a C-terminal FAT domain [64, 79, 93]. The FERM domain is involved in localizing Pyk2 to the plasma membrane and facilitates Pyk2 binding to phosphatidylinositol bisphosphate (PIP2) [94, 95]. Although structural similarities exist between FAK and Pyk2, these proteins appear to exhibit unique effects on adhesion structures in different cells. Recently, it was reported that deletion of FAK in osteoclasts leads to the formation of peripheral podosome belt, whereas deletion of Pyk2 resulted in small podosome rings [96]. In addition, deletion of FAK but not Pyk2, in lung carcinoma CL1-5 cells resulted in decreased formation of podosomes rosettes [96]. These findings suggest that FAK and Pyk2 may regulate different patterning of podosome organization in osteoclasts [96]. Although the mechanism is unknown, the recruitment of downstream effector proteins is likely to be important in the differential roles of these kinases. 

## 4. Invadopodia

Invadopodia appear as dynamic protrusions of the plasma membrane, containing a central actin core surrounded by adhesion proteins, signaling molecules, and scaffolding proteins [59]. In addition, invadopodia are sites of ECM degradation and are often observed in highly migratory metastatic cancer cells [57]. Invadopodia share, overlapping features with podosomes, especially with regards to their intracellular localization, composition of proteins, and cell types in which they are found [55, 59, 62, 97–99] (see Table 1). However, differences between invadopodia and podosomes do exist. In particular, podosomes are short lived (minutes) and found in phagocytic cells such as osteoclasts, whereas invadopdia persist for hours and found primarily in cancer cells [97, 100]. Like podosomes, invadopodia are regulated by a multitude of signaling proteins such as the Src-family kinases, protein kinase C [55, 101–104], cdc42, N-WASP, and Arp2/3 [105, 106]. Dynamin has also been shown to participate in focal extracellular matrix degradation by invasive cells [107]. Although integrin signaling in the initiation of podosome formation is well established, the role of integrins in invadopodia is not yet clear [108]. 

 The life cycle of invadopodia involves initiation, extension, ECM degradation, and disassembly. Each of these steps involves F-actin remodeling and the activation/deactivation of signaling proteins around the central actin core. The initiation of invadopodia is known to be stimulated by EGF, PDGF and reactive oxygen species (ROS) [76]. Following initiation by EGF receptor activation, Src and the tyrosine kinase Abl (Abelson) are recruited and activated [102, 105, 109]. This results in an increase in actin polymerization and cortactin phosphorylation within the elongating invadopodium [102, 105, 109]. Microscopic imaging has shown that cortactin accumulates in invadopodia prior to F-actin nucleation [101], matrix metalloprotease accumulation, and matrix degradation [55], suggesting that cortactin isan early player in this process. In addition to the filamentous actin network, microtubules and intermediate filaments also participate in the elongation and extension of invadopodia [110], with the resulting structure resembling the arrangement of actin filaments in podosomes. The growing protrusive membrane is supplied by vesicular trafficking to sites of invadopodia extension and is controlled by the Golgi apparatus and by F-bar proteins such as CIP4 (cdc42 interacting protein) [107, 111] and the Ena/VASP family protein, Mena [112–114]. Membrane fusion and actin remodeling by dynamin have also been shown to be involved in invadopodia formation [115, 116]. Like podosomes, the formation and stabilization of invadopodia involves microtubule-dependent transport [108, 110]. The third step in the life cycle of invadopodia, and the major function of these structures, is ECM degradation. This function is shared with podosomes but is absent in focal adhesions. ECM degradation is facilitated by secretion of a variety of matrix metalloproteases and serine proteases [56, 112, 117, 118] and is thought to be regulated by cortactin, an actin regulating protein [55]. The secreted proteases act to degrade components of the ECM, thereby facilitating cellular migration and invasion [119, 120]. Finally, the disassembly of invadopodia involves depolymerization of the actin core [121] and has been shown to be regulated by ERK, paxillin, and the calcium-dependent cysteine protease, calpain, which degrades cortactin [121, 122].

 The Src family kinases have been demonstrated to be critical for invadopodia formation and maturation. However, several lines of evidence support a role for Src in focal adhesion and podosome stability [123–125]. Similarly, as discussed above, FAK is important for focal adhesion turnover [126] but deletion of FAK has been also shown to increase invadopodia formation [6, 18] and suppress podosomes rosettes formation n fibroblasts [96]. Moreover, FAK has been shown to regulate a switch from phosphotyrosine-containing proteins at focal adhesions to invadopodia through the temporal regulation of active Src [6]. In the same study, it was shown that FAK-Src signaling also plays a significant role in cancer cell invasion [6, 116]. The apparent overlapping role of FAK and Src in different adhesion structures can be explained by the formation of dynamic proteins complexes between these molecules. For example, the major autophosphorylation site in FAK is Y397 (Y402 in Pyk2) which serves as an SH2-binding site, allowing Src to bind FAK (or Pyk2) [127]. The binding of Src leads to release of its own autoinhibitory catalytic domain, leading to the full activation of Src and to the activation of distinct downstream signaling cascades [128]. 

## 5. Adhesion Proteins and Human Disease

In the following section, and summarized in Table 2, we provide an overview of the role of key focal adhesion proteins and their potential link to human diseases. 

### 5.1. Skeletal Disease

Bones provide structural rigidity to the skeleton and are constantly remodeled to maintain calcium and mineral homeostasis and repair skeletal damage. Bone architectural integrity relies in part on the rate of apoptosis of bone-forming osteoblasts. The activity of Pyk2 is also linked with a variety of metabolic conditions, including the regulation of bone mass. Deletion of Pyk2 leads to increased bone mass in mice [64, 84] due in part to defects in focal adhesion signaling in osteoblasts (our unpublished findings) as well as changes in podosome dynamics in osteoclasts [14, 87]. Src is also important for osteoclast function [129]. Deletion of Src impairs osteoclast bone resorbing activity and mice lacking Src exhibit severe osteopetrosis and exhibit defects in tooth eruption [129]. Studies have shown that disruption of the interaction of *α*-actinin with integrins at focal adhesions increases osteoblast apoptosis, which shifts the balance in favor of osteoclast activity, resulting in bone loss [130].

### 5.2. Role in Cancer

As discussed above, invadopodia formation is tightly linked with cancer metastasis. For example, recently, it was demonstrated that the transcription factor Twist1, a central regulator of the epithelial mesenchymal transition, promotes invadopodia formation through upregulation of platelet-derived growth factor receptor expression and activity, which play significant role in human breast cancer metastasis [120]. Invadopodia formation and therefore cancer invasion also involves the adaptor proteins TKS4 and TKS5 (tyrosine kinase substrate 4 and 5) [131]. It was also found that TKS5 colocalizes to invadopodia in different human cancer cells and that decreased TKS5 expression leads to decreased podosome formation and to reduced tumor metastasis [132]. Thus, TKS4 and TKS5 could potentially be used as therapeutic targets for the treatment of certain types of cancer. Other studies have demonstrated that loss of function of the *Fgd1* gene, which encodes a GTP-exchange factor, was associated with a rare inherited human developmental disease called faciogenital dysplasia. Fgd1 mutations in humans cause skeletal and neurological effects. However, Fgd1 was also shown to be involved in invadopodia biogenesis and ECM degradation [133], and to be expressed in human prostate and breast cancer cells, suggesting it may also be critical for cancer progression and tumorigenesis.

 Several independent studies have demonstrated a critical role for FAK in tumor progression and invasion. Elevated FAK phosphorylation has been observed in several cancers, including breast, colon, thyroid, prostate, oral, neck, and ovarian cancer [134, 135]. Deletion of FAK from tumor cells or breast cancer cells resulted in decreased tumor progression [136, 137], while in endothelial-specific tamoxifen-inducible FAK knockout mice, tumor growth and angiogenesis were reduced [138], indicating that FAK may be important for tumorigenesis. In addition, quantitative real-time PCR has shown an elevation of FAK expression in malignant gastrointestinal stromal tumors [139]. Increased FAK expression was also detected in esophageal squamous cell carcinomas and was associated with cell differentiation, tumor invasiveness, and lymph node metastasis [140]. Also, it was found that FAK was overexpressed in esophageal squamous cell carcinoma which have led to cell differentiation, tumor invasiveness, and lymph node metastasis [140]. *In vitro* evidence also demonstrates that Src-FAK signaling is associated with elevated tumor cell metastases, invadopodia formation, and promotes cell invasion [141, 142]. The Src family of tyrosine kinases are important for embryonic stem cell self-renewal and are key regulators of signal transduction in various cells, including cancer cells [143, 144]. Collectively, these findings provide strong evidence that overexpression of FAK and other proteins localized to invadopodia are important for invadopodia formation and tumor metastasis. Although there is a strong correlation between the expression of FAK and Src in invadopodia and the potential link of these kinases in cancer progression and invasion, it is not yet clear if Src-FAK signaling specifically in invadopodia is critical role for tumor growth. Nevertheless, FAK may be a useful biomarker for cancer cell metastasis and inhibitors to FAK or Src may be useful to limit disease progression [145]. To this end, the FAK inhibitor PND-1186 was found to dramatically decrease FAK activity in breast carcinoma cells, resulting in tumor cell apoptosis [146].

 Lung cancer is considered to be one of the leading causes of mortality among the malignant tumors worldwide. It has been reported that small cell lung cancers (SCLCs) constitute 15–25% of all newly diagnosed primary lung cancers [147]. In the same study, it was shown that inhibition of Pyk2 by lentiviral RNAi or Src using a chemical inhibitor (PP2) reduced SCLC survival and proliferation in liquid culture and in soft agar [147]. In addition, it was demonstrated that Pyk2 also plays an important role in human non-small cell lung cancer (NSCLC) [148]. This was based on the detection of higher levels of Pyk2, as determined by Western blotting and immunohistochemistry, in NSCLC biopsies compared to nontumors [148]. In other studies, FAK signaling was shown to be important in the early stages of mammary adenocarcinoma lung metastasis [149]. It was further demonstrated that the dominant-negative FAK inhibitor, FRNK, blocked lung metastasis if added one day before tumor cell injection, but had no effect if given several days after tumor cell injection [149]. Furthermore, it was demonstrated that depletion of FAK, but not Pyk2, in lung carcinoma CL1-5 cells, decreased the formation of podosome rosette structures and decreased cell invasion [96]. Nevertheless, despite strong *in vitro* and *ex vivo* evidence linking FAK, Pyk2, and Src to various cancers, a direct link between kinase activity, effects on podosome/invadopodia formation, and cancer cell metastasis/function is currently lacking. 

 Several studies also suggest a link between the adhesion kinases and prostate cancer. For example, it has been shown that metastatic prostate cancer cells express elevated FAK mRNA levels and protein phosphorylation [150]. More recent studies also suggest that inhibition of Pyk2 and FAK may be an important therapeutic strategy to decrease prostate cancer progression [151]. Sun et al. used a mouse xenograft model injected with a chemical inhibitor of FAK and Pyk2 (PF-562,271) [151]. After two weeks of treatment with PF-562,271 (25 mg/kg), the mouse xenograft model showed a 62% decrease in tumor growth, compared to control mice [151]. Leupaxin was found to associate with Pyk2, c-Src, and PTP-PEST. *In vitro* studies also suggest that the migration of prostate cancer cells (PC-3) may be regulated by protein complexes involving leupaxin, Pyk2, and the tyrosine phosphatase PTP-PEST, which dephosphorylates Pyk2 [91, 152]. Furthermore, it was shown that invasion of PC3 cells in a gelatin matrix is controlled by invadopodia and ECM degradation [153]. 

 Astrocytomas represent the most common intracranial neoplasms accounting for 60% of all primary brain tumors. In separate studies, FAK and Pyk2 expressions have been shown to elevated in human brain astrocytomas [154–156]. In addition, a novel kinase inhibitor of FAK (TAE226) has been shown to increase tumor cell apoptosis in brain tumors [157]. Finally, others have demonstrated that administration of Src family kinase inhibitors, PP1 and Dasatinib, results in a dramatic increase in apoptosis of several pediatric brain tumor cell lines, compared to control cell lines was observed [158]. Collectively, the above findings suggest that inhibition of Pyk2 and FAK and other signaling molecules impair tumor migration by blocking the biogenesis of invadopodia which are important for ECM degradation. 

### 5.3. Pulmonary and Other Diseases

Pyk2 was identified as a central regulator for angiogenesis of pulmonary vascular endothelial cells [159]. Additional studies show that Pyk2 is essential in regulating airway inflammation, Th2 cytokine secretion, and airway hyper-responsiveness in ovalbumin-sensitized mice during antigen challenge *in vivo* [160]. Inhibition of Pyk2 blocked broncho-alveolar lavage, eosinophilia, mucous gland hyperplasia, and airway hyper-responsiveness, conditions that are also characteristic of the asthmatic state in humans. In addition, deletion of Pyk2 leads to developmental defects, abnormal macrophage activity, obesity, and insulin resistance under a high-fat diet [161, 162]. Pyk2 activity in the heart may also protect against arrhythmia [163]. Although the mechanism by which Pyk2 regulates these physiological processes is still unknown, therapeutic strategies that target Pyk2 might be a novel approach for the treatment of a variety of metabolic and pathological diseases. Finally, it has been shown that dynamin mutations are associated with human centronuclear myopathy and Charcot-Marie-Tooth neuropathy [164–166]. These diseases are currently attributed to defects in dynamin-mediated endocytosis. However, it is interest to note that dynamin plays an important role in actin remodeling, which is linked to its function in membrane endocytosis [115, 116]. Therefore, it is possible that dynamin's role in actin remodeling and adhesion structure turnover [43, 44, 63, 92, 107] may also be involved in these pathologies, although this remains to be determined. 

## 6. Summary and Perspectives

In summary, focal adhesions, podosomes, and invadopodia facilitate adhesion to the matrix and cellular migration. In addition to adhesion, podosomes and invadopodia have evolved the unique function of ECM degradation. The focal adhesion kinases, FAK and Pyk2, exhibit overlapping and unique roles in the biogenesis, stability, and disassembly of these different adhesion structures. There is currently a growing body of evidence linking these and other kinases to the biogenesis of different adhesion structures. In addition, a great deal of studies suggests a link between the expression levels of these kinases and several human diseases, especially cancer (see Table 2). Finally, emerging evidence suggests that disrupting the activity of the adhesion kinases not only disrupts the formation of the adhesion structures, but it may also be useful in the treatment of serious medical conditions such as cancer and osteoporosis. A greater understanding of the function of adhesion kinases and the adhesion structures they control will offer future avenues for therapeutic interventions against several human diseases.

## Figures and Tables

**Table 1:** Common and unique features of focal adhesions, podosomes, and invadopodia. See text for details.

	Focal adhesion	Podosomes	Invadopodia
Appearance	dense plaques of F-actin	F-actin bundle core surrounded by actin cloud	F-actin bundle core surrounded by actin cloud

Size	width: 2–6 *μ*m	width: 0.5–2 *μ*m length: 0.5–2 *μ*m	width: 0.5–2 *μ*m length: >2 *μ*m

Duration (half-life)	hours	minutes	hours

Cell expression	numerous nonmigrating fibroblastic cells	monocytic cells osteoclasts endothelial cells smooth muscle cells Src-transformed fibroblasts	carcinoma cells Src-transformed fibroblasts

Location	often found at leading edge of cell	ventral side of the cellular membrane	ventral side of the cellular membrane

Extracellular matrix degradation	no	yes	yes

Common signaling molecules	focal adhesion proteins GTPases actin regulators motor proteins tyrosine kinases phosphatases scaffolding molecules	focal adhesion proteins GTPases actin regulators motor proteins tyrosine kinases phosphatases scaffolding molecules	focal adhesion proteins GTPases actin regulators motor proteins tyrosine kinases phosphatases scaffolding molecules

Distinct Features	integrin receptors	Integrin receptors matrix-degrading enzymes	matrix-degrading enzymes

**Table 2:** Signaling proteins and their link to adhesion structures and disease. ^+^Humans mutations are associated with disease. Mutations in dynamin are linked to centronuclear myopathy and Charcot-Marie-Tooth neuropathy in humans. ^∗^Bone mass regulation is based on knockout mice studies. Other disease indications are predicted based on animal studies and *in vitro* studies. n/d: not determined. See text for details.

Adhesion protein	Cell type	Adhesion structure	Disease indication
FAK	osteoblasts	focal adhesions	regulation of bone density^∗^
	osteoclasts	podosomes	regulation of bone density^∗^
	endothelial cells	podosomes?	angiogenesis
	lung carcinoma cells	podosomes	cancer metastasis
	various cancer cells	invadopodia	cancer metastasis

Pyk2	osteoblasts	focal adhesions	regulation of bone density^∗^
	osteoclasts	podosomes	regulation of bone density^∗^
	endothelial cells	podosomes?	angiogenesis
	various cancer cells	invadopodia	cancer metastasis

Src	osteoblasts	focal adhesions	regulation of bone density^∗^
	osteoclasts	podosomes	regulation of bone density^∗^
	various cancer cells	invadopodia	cancer metastasis

Dynamin	fibroblasts	focal adhesions	n/d
	osteoblasts	focal adhesions	regulation of bone density^∗^
	osteoclasts	podosomes	regulation of bone density^∗^
	neurons	n/d	neuropathy^+^

Twist1	epithelial cells	invadopodia	cancer metastasis

TKS4/5	human cancer cells	invadopodia	cancer metastasis

Leupaxin	osteoclasts	podosomes	regulation of bone density^∗^
	cancer cells	invadopodia	cancer metastasis

Fgd1	osteoblasts	focal adhesions?	skeletal abnormalities^+^
	cancer cells	invadopodia	prostate and breast cancer metastasis

## Abbreviations

ECM: Extracellular matrix
OC: Osteoclast
OB: Osteoblast
FAK: Focal adhesion kinase
SCLC: Small cell lung cancer
NSCLC: Nonsmall cell lung cancer.
